# Net growth rate of continuum heterogeneous biofilms with inhibition kinetics

**DOI:** 10.1038/s41522-017-0045-y

**Published:** 2018-03-08

**Authors:** Elio Emilio Gonzo, Stefan Wuertz, Veronica B. Rajal

**Affiliations:** 1 INIQUI (CONICET)—Facultad de Ingeniería, Universidad Nacional de Salta, Av. Bolivia 5150, Salta, 4400 Argentina; 2 Singapore Centre for Environmental Life Sciences Engineering (SCELSE), Nanyang Technological University, Singapore, 637551 Singapore; 3 School of Civil and Environmental Engineering, Nanyang Technological University, Singapore, 639798 Singapore

## Abstract

Biofilm systems can be modeled using a variety of analytical and numerical approaches, usually by making simplifying assumptions regarding biofilm heterogeneity and activity as well as effective diffusivity. Inhibition kinetics, albeit common in experimental systems, are rarely considered and analytical approaches are either lacking or consider effective diffusivity of the substrate and the biofilm density to remain constant. To address this obvious knowledge gap an analytical procedure to estimate the effectiveness factor (dimensionless substrate mass flux at the biofilm-fluid interface) was developed for a continuum heterogeneous biofilm with multiple limiting-substrate Monod kinetics to different types of inhibition kinetics. The simple perturbation technique, previously validated to quantify biofilm activity, was applied to systems where either the substrate or the inhibitor is the limiting component, and cases where the inhibitor is a reaction product or the substrate also acts as the inhibitor. Explicit analytical equations are presented for the effectiveness factor estimation and, therefore, the calculation of biomass growth rate or limiting substrate/inhibitor consumption rate, for a given biofilm thickness. The robustness of the new biofilm model was tested using kinetic parameters experimentally determined for the growth of *Pseudomonas putida* CCRC 14365 on phenol. Several additional cases have been analyzed, including examples where the effectiveness factor can reach values greater than unity, characteristic of systems with inhibition kinetics. Criteria to establish when the effectiveness factor can reach values greater than unity in each of the cases studied are also presented.

## Biofilm growth: Investigating inhibition

A method to model and assess the effect of interactions and processes that inhibit biofilm growth closes a gap in understanding. Biofilm growth is often inhibited by chemicals used or produced by microorganisms, or by other components and characteristics of the surroundings. This has been a neglected aspect of the procedures used to analyze and predict biofilm behavior. Stefan Wuertz at Nanyang Technological University in Singapore, with colleagues in Singapore and Argentina, mathematically modeled these often-overlooked inhibitory effects. They tested the accuracy of their procedure on complex biofilms under a variety of growing conditions. The insights gained into growth inhibition should help with the design of bioreactors, including those used to degrade pollutants. The method should be widely applicable to all types of biofilms, ranging from laboratory to industrial scale applications.

## Introduction

The successful design of large-scale bioreactors for pollutant degradation requires the ability to accurately predict the growth of microorganisms. A great number of theoretical investigations of the growth rate of one-species or mixed species biofilms have focused on the difficulty of transferring substrates and products between the fluid phase and the cells inside a biofilm. These analyses were based on the theory of mass transport and diffusion in porous media but rarely considered inhibition. In contrast, many experimental investigations have reported inhibition kinetics of different types, e.g., substrate inhibition,^1–4^ product inhibition,^5–7^ substrate and product inhibition,^8,9^ inhibition by other compounds present in the fluid phase,^10^ inhibition effects of heavy metals ions,^11^ and wastewater treatment under salt-affected conditions,^12^ among others. Equations were usually fitted to experimental data in all of these field or laboratory studies, without developing predictive scenarios of what would happen in different situations (for example, as a result of product inhibition), and only some specifically addressed biofilm systems.^5,11^ Modeling studies on substrate utilization and inhibition effects in biofilms are few and far between.^13–16^ It is, therefore, of interest to model the effect of the coupled processes of substrate and product diffusion on the net rate of biofilm growth when different types of inhibition mechanisms are considered.

The objectives in the present study were to extend a procedure to estimate analytically the effectiveness factor (dimensionless substrate mass flux at the biofilm-fluid interface) for a continuum heterogeneous biofilm with multiple limiting substrates Monod kinetics^17^ to different types of inhibition kinetics. We tested a variety of scenarios when (i) the substrate is the limiting component, (ii) the inhibitor is the limiting species, or (iii) the substrate is also the inhibitor. The model describes a heterogeneous biofilm with variable distribution of biofilm density, activity, and effective diffusivity.

## Model development

### Inhibition modeling

The effect of an inhibitory component on the specific growth rate, *r*, is given by:^18,19^

1 $$r = q_{{\mathrm{max}}}\left( {\frac{{C_{\rm A}}}{{K_{\rm A} + C_{\rm A}}}} \right)\left( {\frac{{K_{\rm I}}}{{K_{\rm I} + C_{\rm I}}}} \right)$$

where *q*_max_ is the maximum specific growth rate, *C*_A_ is the concentration of the key substrate A, *K*_A_ is the Monod half rate constant for substrate A, *C*_I_ is the concentration of the inhibitor I, and *K*_I_ is the concentration of the inhibitor resulting in 50% inhibition of the maximum rate (all variables are described in detail in Box 1).

Taking into account that both the effective diffusivities of substrate and inhibitory component and the biofilm density vary with the position (*x*) in the biofilm, the mass balances for the substrates and inhibitors, at steady state (pseudo-steady state), are given by:

2 $$\frac{{\rm d}}{{{\rm d}x}}D_{{\rm fA}}(x)\frac{{{\rm d}C_A}}{{{\rm d}x}} = \frac{{q_{{\mathrm{max}}}}}{{Y_{\rm A}}}X_{\rm f}(x)\left( {\frac{{C_{\rm A}}}{{K_{\rm A} + C_{\rm A}}}} \right)\left( {\frac{{K_{\rm I}}}{{K_{\rm I} + C_{\rm I}}}} \right),$$

3 $$\frac{{\rm d}}{{{\rm d}x}}D_{{\rm fI}}(x)\frac{{{\rm d}C_{\rm I}}}{{{\rm d}x}} = \frac{{q_{{\mathrm{max}}}}}{{Y_{\rm I}}}X_{\rm f}(x)\left( {\frac{{C_{\rm A}}}{{K_{\rm A} + C_{\rm A}}}} \right)\left( {\frac{{K_{\rm I}}}{{K_{\rm I} + C_{\rm I}}}} \right),$$

where *D*_fA_ and *D*_fI_ are the effective diffusivities of the substrate and inhibitor, *X*_f_ is the biofilm density, and *Y*_A_ and *Y*_I_ are the biomass yield coefficients of the key substrate A and inhibitor I, respectively.

The following assumptions are made in Eqs. (2) and (3):

- The biofilm is a continuum.
- Substrate and inhibitor are transferred by diffusion only, in accordance with Fick’s law.^20^
- Microorganisms consume the substrate and inhibitor at a rate according to Double-Monod inhibition kinetics.
- Biofilm density and substrate effective diffusivity change in the *x* direction.
- Steady state (pseudo-steady state) conditions apply and the rate of substrate consumption is higher compared to the rate of biofilm growth.
- External resistances to mass transfer are neglected. Therefore, the substrate and inhibitor concentrations at the biofilm-fluid interphase, *C*_As_ and *C*_Is_, respectively, are known.
- The substratum where the biofilm grows is impermeable.

Under these assumptions, the appropriate boundary conditions for Eqs. (2) and (3) are:

4 $${\mathrm{At}}\quad x = L_{\rm f}\quad C_{\rm A} = C_{\rm {As}}\quad C_{\rm I} = C_{\rm {Is}}$$

5 $${\mathrm{At}}\quad x = 0\quad \frac{{{\rm d}C_{\rm A}}}{{{\rm d}x}} = 0\quad {\mathrm{and}}\quad \frac{{{\rm d}C_{\rm I}}}{{{\rm d}x}} = 0$$

with *L*_f_ representing the average biofilm thickness.

**Fig. 1:**
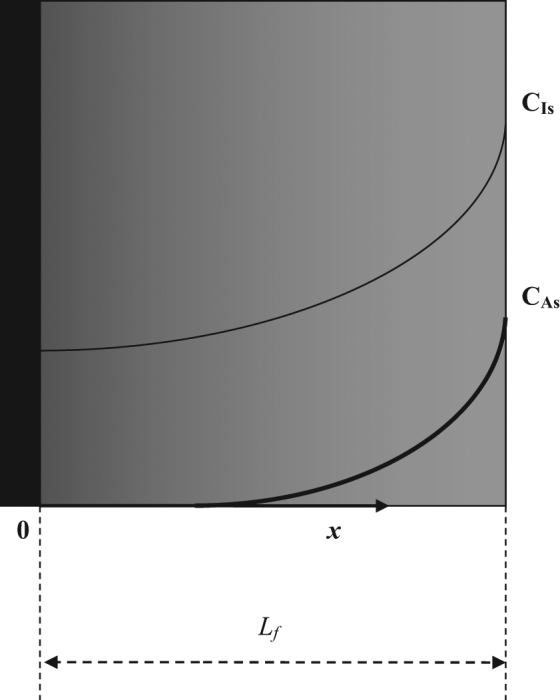
Schematic representation of the continuum heterogeneous biofilm model with concentration profiles for substrate A and inhibitory component I. *C*_As_ concentration of substrate (A) at the surface of the biofilm, kg/m^3^, *C*_Is_ concentration of inhibitor (I) at the surface of the biofilm, kg/m^3^, *L*_f_ biofilm thickness. In black: impermeable substratum

The concentrations of the substrate, A, and inhibitory component, I, vary as a function of biofilm depth in the continuous heterogeneous biofilm (Fig. 1). The *D*_fA_*(x)*, *D*_fI_*(x)*, and *X*_f_*(x)* profiles and dimensionless differential equations describing the system can be found in Supplementary Information 1.

### Case study

We studied three possible scenarios:

Case (a): If the substrate (A) is the limiting component rather than the inhibitor, which can be a reaction product, then

6 $${\mathrm{\Gamma }}_{\rm A} = 1\quad {\mathrm{\Gamma }}_{\rm I} = \nu _{\rm I}\frac{{C_{{\rm As}}\,\overline D _{\rm {fA}}}}{{C_{{\rm Is}}\,\overline D _{{\rm fI}}}}$$

with

7 $$C_{\rm I}^ \ast = {\mathrm{\Gamma }}_{\rm I}\left( {C_{\rm A}^ \ast - 1} \right) + 1$$

Therefore, Eq. (S1–14) results in (see Supplementary Information 1):

8 $$r_{\mathrm A}^ \ast = \left( {\beta _{\mathrm A} + 1} \right)\left( {\beta _{\mathrm I} + 1} \right)\frac{{C_{\mathrm A}^ \ast }}{{\left( {\beta _{\mathrm A} + C_{\mathrm A}^ \ast } \right)}}\frac{1}{{\left[ {\beta _{\mathrm I} + {\mathrm{\Gamma }}_{\mathrm I}\left( {C_{\mathrm A}^ \ast - 1} \right) + 1} \right]}}$$

and

9 $$\left( {\frac{{dr_{\rm A}^ \ast }}{{dC_{\rm A}^ \ast }}} \right)_{C_{\rm A}^ \ast = 1} = r_{\rm A}^{ \ast \prime }(1) = \frac{{\beta _{\rm A}}}{{(\beta _{\rm A} + 1)}} - \frac{{{\mathrm{\Gamma }}_{\rm I}}}{{(\beta _{\rm I} + 1)}}$$

Case (b): If the inhibitor is the limiting substrate, rather than the substrate, then

10 $${\mathrm{\Gamma }}_{\rm A} = \nu _{\rm A}\frac{{C_{{\rm Is}}\,\overline D _{{\rm fI}}}}{{C_{{\rm As}}\,\overline D _{{\rm fA}}}}\quad {\mathrm{and}}\quad {\mathrm{\Gamma }}_{\rm I} = 1$$

with

11 $$C_{\rm A}^ \ast = {\mathrm{\Gamma }}_{\rm A}\left( {C_{\rm I}^ \ast - 1} \right) + 1$$

Therefore, Eq. (S1–14) results in

12 $$r_I^ \ast = \left( {\beta _{\rm A} + 1} \right)\left( {\beta _{\rm I} + 1} \right)\frac{{\left[ {{\mathrm{\Gamma }}_{\rm A}\left( {C_{\rm I}^ \ast - 1} \right) + 1} \right]}}{{\left[ {\beta _{\rm A} + {\mathrm{\Gamma }}_{\rm A}\left( {C_{\rm I}^ \ast - 1} \right) + 1} \right]}}\frac{1}{{\left( {\beta _{\rm I} + C_{\rm I}^ \ast } \right)}}$$

and

13 $$\left( {\frac{{{\rm d}r_{\rm I}^ \ast }}{{{\rm d}C_{\rm I}^ \ast }}} \right)_{C_{\rm I}^ \ast = 1} = r_{\rm I}^{ \ast \prime }(1) = \frac{{{\mathrm{\Gamma }}_{\rm A}\,\beta _{\rm A}}}{{\left( {\beta _{\rm A} + 1} \right)}} - \frac{1}{{\left( {\beta _{\rm I} + 1} \right)}}$$

Case (c): This is a special situation of case (b), where there is a single substrate that is also the inhibitor.

14 $${\mathrm{\Gamma }}_{{\rm AI}} = 1$$

The dimensionless kinetic rate expression in this case is (see Eq. (S1–14)):

15 $$r_{{\rm AI}}^ \ast = \left( {\beta _{\rm A} + 1} \right)\left( {\beta _{{\rm AI}} + 1} \right)\frac{{C_{\rm A}^ \ast }}{{\left( {\beta _{\rm A} + C_{\rm A}^ \ast } \right)}}\frac{1}{{\left( {\beta _{{\rm AI}} + C_{\rm A}^ \ast } \right)}}$$

with

16 $$\it \beta _{{\rm AI}} = \frac{{{\rm \it K_I}}}{{C_{{\rm As}}}}$$

17 $$\left( {\frac{{{\rm d}r_{{\rm AI}}^ \ast }}{{{\rm d}C_{\rm A}^ \ast }}} \right)_{C_{\rm A}^ \ast = 1} = r_{{\rm AI}}^{ \ast \prime }(1) = \frac{{\beta _{\rm A}}}{{\left( {\beta _{\rm A} + 1} \right)}} - \frac{1}{{\left( {\beta _{{\rm AI}} + 1} \right)}}$$

#### Mass balance differential equations

The mass balance differential equations for the corresponding limiting component (in each case), considering valid Eq. (S1–13), are:

Case (a):

18 $$\frac{{\rm d}}{{{\rm d}x^ \ast }}D_{{\rm fA}}^ \ast \frac{{{\rm d}C_{\rm A}^ \ast }}{{{\rm d}x^ \ast }} = \phi ^2\,X_{\rm f}^ \ast \,r_{\rm A}^ \ast$$

Case (b):

19 $$\frac{d}{{dx^ \ast }}D_{fI}^ \ast \frac{{dC_I^ \ast }}{{dx^ \ast }} = \phi ^2\,X_f^ \ast \,r_I^ \ast$$

Case (c):

20 $$\frac{{\rm d}}{{{\rm d}x^ \ast }}D_{{\rm fA}}^ \ast \frac{{{\rm d}C_{\rm A}^ \ast }}{{{\rm d}x^ \ast }} = \phi ^2\,X_{\rm f}^ \ast \,r_{{\rm AI}}^ \ast$$

### Particular conditions

Analyzing the asymptotic solutions of the different cases studied for *ϕ*^*2*^ ≪ 1 (see Supplementary Information 2), there is a condition under which the first derivative of r*, evaluated at $C_{\rm A}^ \ast$ = 1, is negative. In this situation the value of parameter *σ* (*σ*_A_*, σ*_i_), will be negative. This is a necessary but not sufficient condition to assure that there will be a region of the *η* versus *ϕ* curve, in which *η* values will be greater than one.

These particular situations are met in the following scenarios below.

Case (a): Substrate A is the limiting substrate and the inhibitor is another reactant, as in Eq. (9). The effectiveness factor, *η* (the ratio between the diffusion-limited substrate consumption rate and the substrate consumption rate that is not limited by diffusion), can reach values greater than unity when:

21 $$\frac{{{\mathrm{\Gamma }}_{\rm I}}}{{\beta _{\rm I} + 1}} > \frac{{\beta _{\rm A}}}{{\beta _{\rm A} + 1}}$$

Case (b): The inhibitor is the limiting component, as in Eq. (13). Then:

22 $$\frac{1}{{\beta _{\rm I} + 1}} > \frac{{{\mathrm{\Gamma }}_{\rm A}\beta _{\rm A}}}{{\beta _{\rm A} + 1}}$$

Case (c): The same component is both substrate and inhibitor, as in Eq. (17).

23 $$\frac{1}{{\beta _{{\rm AI}} + 1}} > \frac{{\beta _{\rm A}}}{{\beta _{\rm A} + 1}}\quad {\mathrm{or}}\quad \beta _{{\rm AI}} < \frac{1}{{\beta _{\rm A}}}$$

In all these cases, the matching expressions (S2–18), (S2–22) and (S2–26) are able to predict the true value of the effectiveness factor. When *η* is greater than one, the diffusion limited substrate consumption rate is greater than the rate without diffusion resistance.

## Results and discussion

### Model setup

The continuum heterogeneous model was applied to different inhibition scenarios:

Case (a): The substrate A is the limiting substrate and the inhibitor is another reactant, which can be considered in two different ways:

Case (a_1_): Double Monod inhibition kinetics where substrate A is the limiting component and substrate I the inhibitor.

Case (a_2_): Double Monod inhibition kinetics where substrate A is the limiting component and the inhibitor I is a product.

Case (b): Double Monod inhibition kinetics where the substrate I is the limiting component and reacts with substrate A.

Case (c): Monod inhibition kinetics where the single component A is both substrate and the inhibitor.

**Fig. 2:**
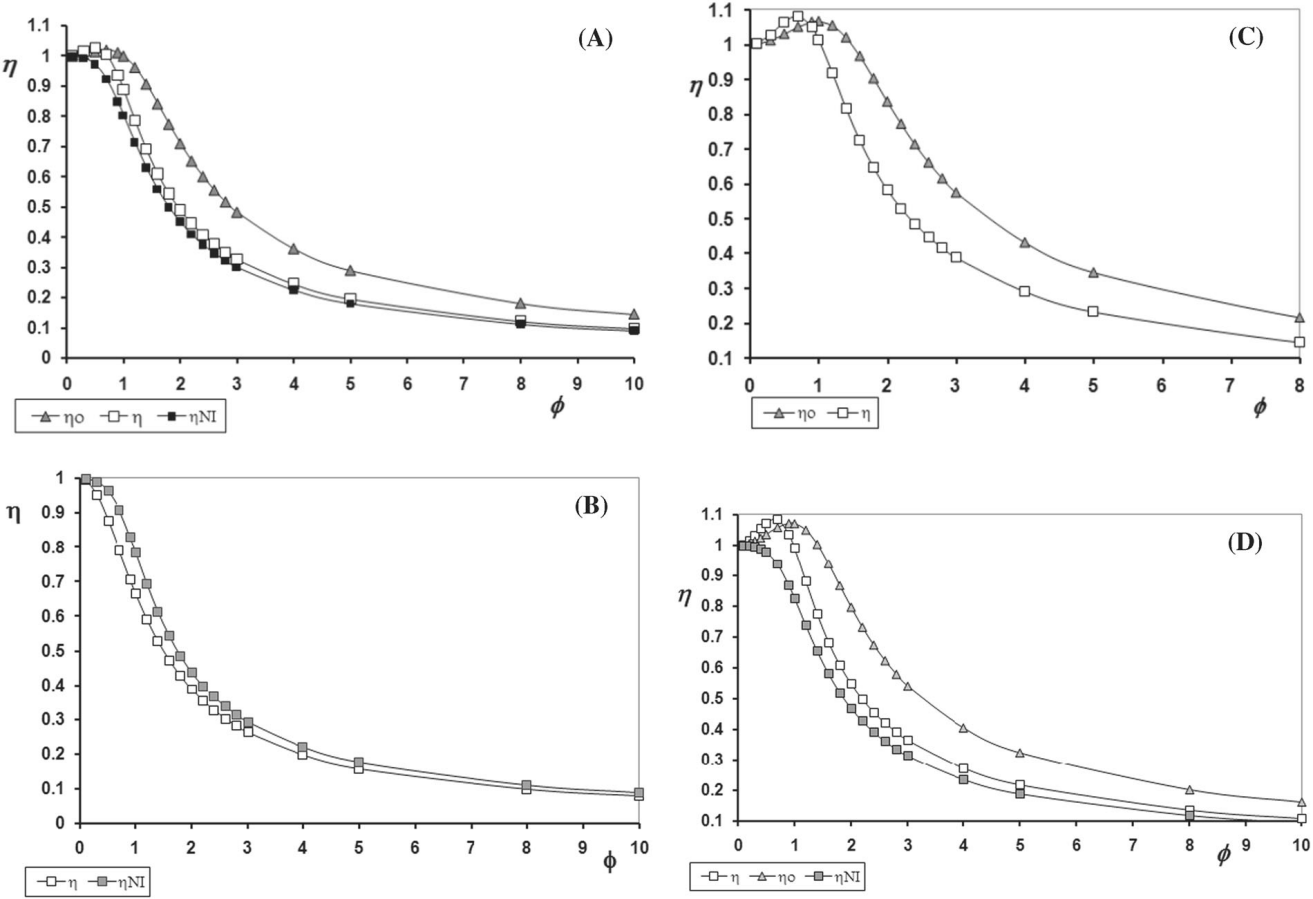
Effectiveness factor for double Monod kinetics with inhibition. *Ψ* = 0.5, *κ* = 4. **a** Case (a_1_): Substrate A is the limiting component; *β*_A_ = 0.05, *β*_I_ = 1.25, Γ_I_ = 0.7. **b** Case (a_2_): The product acts as inhibitor *β*_A_ = 0.1, *β*_BI_ = 1, Γ_B_ = −3. **c** Case (b): The inhibition compound is the limiting component; *β*_A_ = 0.05, *β*_I_ = 1.25, Γ_A_ = 0.7. **d** Case (c): The same component is substrate and inhibitor; *β*_A_ = 0.01, *β*_AI_ = 1. *η*: effectiveness factor for a continuum heterogeneous biofilm with inhibition; *η*_o_: effectiveness factor for a homogeneous system with inhibition; *η*_NI_: effectiveness factor for heterogeneous biofilm without inhibition (single Monod kinetics)

Each case was solved under conditions (parameter values) where effectiveness factor values greater than one could be obtained, except for case (a_2_), which does not have this possibility (see below and Fig. 2b). The effectiveness factor as a function of the Thiele modulus (which is the ratio of the time scale of reaction to the time scale of diffusion) for case (a_1_) considering a continuum heterogeneous biofilm, *η*, a homogeneous biofilm, *η*_0_, and a continuum heterogeneous biofilm without inhibition, *η*_NI_, was considered (Fig. 2a). The possibility of finding values of *η* greater than one is predicted by the condition established by Eq. (21):

$$\frac{{{\mathrm{\Gamma }}_{\rm I}}}{{\beta _{\rm I} + 1}} > \frac{{\beta _{\rm A}}}{{\beta _{\rm A} + 1}}\quad {\mathrm{in}}\,{\mathrm{this}}\,{\mathrm{case}}\quad \frac{{0.7}}{{2.25}} = 0.311 > \frac{{0.05}}{{1.05}} = 0.048$$

For case (a_2_) the inhibitor is a process product. In this case, *η* values greater than one cannot be found because $r_{\rm A}^{ \ast \prime }(1) = \frac{{\beta _{\rm A}}}{{(\beta _{\rm A} + 1)}} - \frac{{{\mathrm{\Gamma }}_{\rm I}}}{{\left( {\beta _{\rm I} + 1} \right)}}$ will always be positive, since ${\mathrm{\Gamma }}_I$ is always negative, due to the fact that *ν*_I_ will be negative (Fig. 2b).

For Double Monod inhibition kinetics where the inhibitor is the limiting component, as in case (b), it is observed that in a continuum heterogeneous biofilm with Thiele modulus values up to 1, the effectiveness factor has values greater than one (maximum ≈ 1.1) (Fig. 2c). This case also obeys the criterion that establishes the condition to find *η* > 1.

$$\frac{1}{{\beta _{\rm I} + 1}} > \frac{{{\mathrm{\Gamma }}_{\rm A}\beta _{\rm A}}}{{\beta _{\rm A} + 1}}\quad {\mathrm{with}}\quad \beta _{\rm A} = 0.05\quad {\mathrm{\Gamma }}_{\rm A} = {\mathrm{ }}0.7\quad \beta _{\rm I} = 1.25$$

Therefore, 0.8 > 0.033.

Finally, Fig. 2d shows the effectiveness factor for case (c), Double Monod inhibition kinetics with a single substrate that is also the inhibitor. The parameters values were chosen in such a way to meet conditions that allow the possibility to find values of *η* > 1. Values of *η* as high as 1.1 were found. For comparison purposes only, the effectiveness factors for a homogeneous biofilm, *η*_0_, and a continuum heterogeneous biofilm without inhibition, *η*_NI_, (Single Monod kinetics) are shown. As expected, the criterion to find *η* values greater than one is also obeyed.

$$\beta _{{\rm AI}} < \frac{1}{{\beta _{\rm A}}}\quad 1 < \left( {1{\mathrm{/}}0.01} \right) = 100$$

### Experimental validation

To assess the robustness of the new biofilm model with inhibition Monod kinetics, a test case was chosen where the substrate acts as nutrient as well as inhibitor (Table 1). The kinetic parameters were taken from the work of Chung et al.^21^ who studied the growth of *Pseudomonas putida* CCRC 14365 during the biodegradation of phenol. The authors used the Haldane inhibition kinetics expression, which is a particular situation of the more general Monod kinetics. It therefore coincides with the Monod kinetics used in the present study, since the ratio between the substrate saturation constant and the inhibition constant (*K*_P_*/K*_PI_) is small (≈0.08).^11^ The Monod growth rate with inhibition is given by Eq. (1). Developing the equation further, it is found that

24 $$r = q_{{\mathrm{max}}}\frac{{C_{\rm P}}}{{K_{\rm P} + C_{\rm P}\left( {1 + \frac{{K_{\rm P}}}{{K_{{\rm PI}}}}} \right) + \frac{{C_{\rm P}^2}}{{K_{{\rm PI}}}}}}$$

When (*K*_P_*/K*_PI_) ≪ 1, then Eq. (24) becomes

25 $$r = q_{{\mathrm{max}}}\frac{{C_{\rm P}}}{{K_{\rm P} + C_{\rm P} + \frac{{C_{\rm P}^2}}{{K_{{\rm PI}}}}}}$$

**Table 1:** Parameters used for the test case of a continuum heterogenous biofilm model of *Pseudomonas putida* growth on phenol^a^

Model Parameter	Designation	Value
Maximum specific growth rate	q_max_	0.38 h^−1^
Phenol^b^ saturation constant	*K*_P_	18.3 g of P m^−3^
Phenol inhibition constant	*K*_PI_	214.5 g of P m^−3^
Biomass yield	*Y*_P_	0.627 g of biomass g P^−1^
Physical properties		
Phenol average diffusion coefficient	$\bar D_{{\rm fP}}$	2.7 × 10^−6^ m^2^ h^−1^
Average value of biomass density in biofilm	$\bar X_{\rm f}$	10,000 g of biomass/m^3^ of biofilm
Biofilm thickness	*L*_f_	75, 150 or 300 μm
Biofilm heterogeneity	*Ψ*	0.5
Relation *D*_wP_*/α*_P_	*κ*	4

Kinetic model: $${\mathrm{r}}_{\mathrm{b}} = {\mathrm{q}}_{{\mathrm{max}}}\,{\mathrm{X}}_{\mathrm{f}}\frac{{{\mathrm{C}}_{\mathrm{P}}}}{{{\mathrm{K}}_{\mathrm{P}} + {\mathrm{C}}_{\mathrm{P}}}}\frac{{{\mathrm{K}}_{{\mathrm{PI}}}}}{{{\mathrm{K}}_{{\mathrm{PI}}} + {\mathrm{C}}_{\mathrm{P}}}}\quad \left[ {\frac{{{\mathrm{g}}\,{\mathrm{biomass}}}}{{{\mathrm{m}}_{{\mathrm{biofilm}}}^3\,{\mathrm{h}}}}} \right]$$

Microbial kinetics at 30 °C

^a^Experimental data taken from Chung et al.^21^

^b^Phenol abbreviated to P

This Eq. (25) is the Haldane equation for substrate inhibition. In contrast to our study, Chung et al.^21^ assumed a biofilm with a constant average density, $\overline X _{\rm f}$, and a constant average substrate effective diffusivity, $\overline D _{{\rm fP}}$. In the present continuum heterogeneous biofilm model, the dimensionless effective diffusivity of phenol and biofilm density change with biofilm position ($x^{\ast}$) as:

26 $$D_{{\rm fP}}^ \ast \left( {x^ \ast } \right) = 0.5\left( {1 + 2\,x^ \ast } \right)$$

27 $$X_{\rm f}^ \ast = - 1.1980 + 3.53266\left( {1 + 2\,x^ \ast } \right)^{ - 0.7782}$$

Therefore, the average biofilm density, $\overline X _{\rm f}$, and the average substrate effective diffusivity, $\overline D _{{\rm fP}}$, are those indicated in Table 1.

### Effect of biofilm thickness

**Fig. 3:**
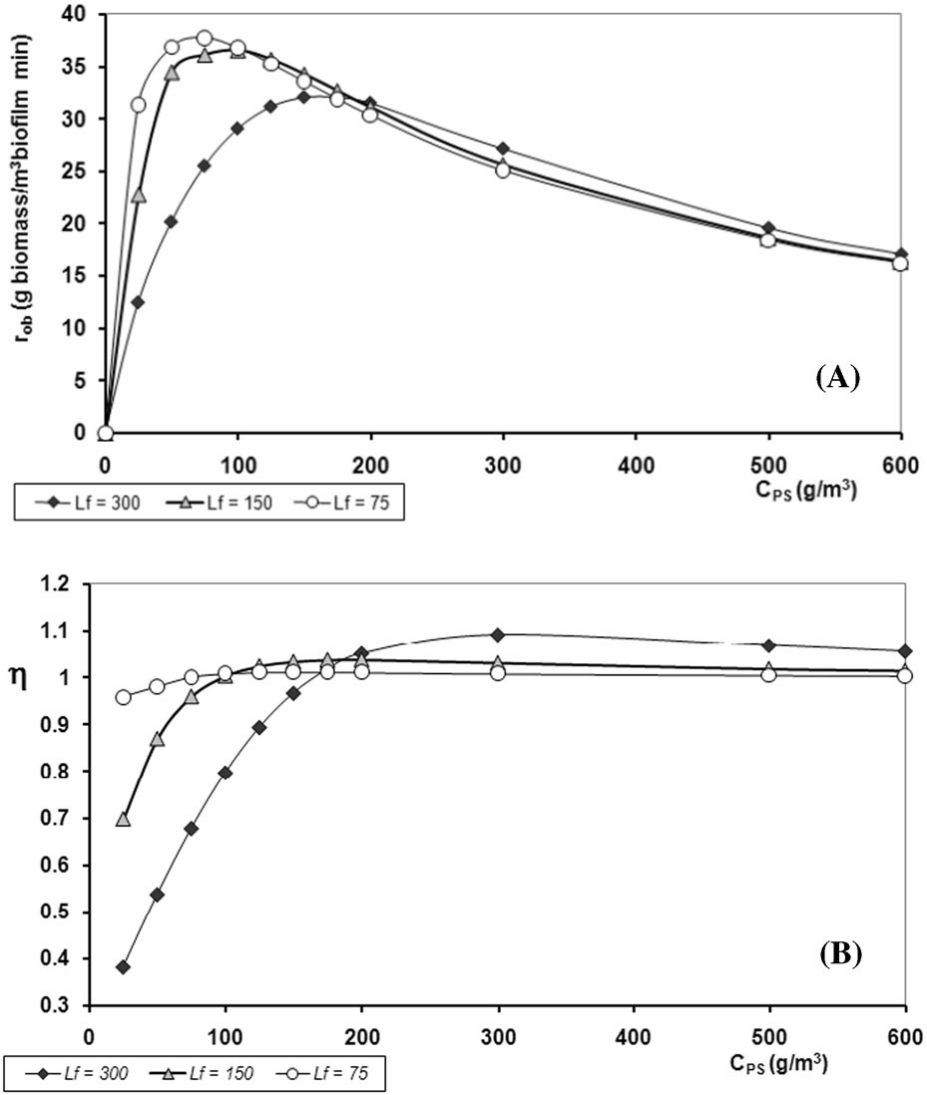
Test case: Model simulation when the substrate is also the inhibitor. Experimental data taken from Chung et al.^21^ **a** Net rate of biomass production, *r*_ob_, and **b** effectiveness factor, *η*, as a function of substrate biofilm-surface concentration, *C*_PS_, for different biofilm thicknesses, *L*_f_, of 75, 150 and 300 μm

Three cases were considered with varying biofilm thicknesses of 75, 150 and 300 μm. All the equations needed for solving the cases are given in Table 2. The net rate of biomass formation was predicted by the continuous heterogeneous biofilm model as a function of phenol concentration, *C*_Ps_, at the biofilm-fluid interphase (Fig. 3a). The effectiveness factor, net rate of phenol consumption, and biomass production for phenol bulk concentrations in the range of 25 to 600 g/m^3^ were calculated using the values of the fundamental parameters given in Table 2. Initially, the biomass production rate increased very fast with increasing phenol concentration until a maximum was reached followed by a slow decrease (Fig. 3a). This behavior is in agreement with experimental findings.^1,21^ The maximum rate was reached at lower phenol concentrations as the biofilm thickness decreased. However, for high phenol concentrations the thicker biofilm produced more biomass than the thinner one. The increase in the rate at a phenol concentration, *C*_Ps_ < *50* g/m^3^, was due to the fact that the rate tends towards a global first order reaction with respect to phenol concentration. The inhibition effect ([*K*_PI_ */(K*_PI_ *+* *C*_Ps_*)*] ≈ 1) was very small. However, when the phenol concentration was high (*C*_Ps_ greater than the substrate inhibition constant) the rate tended toward a negative reaction order (−1) with respect to phenol concentration. In other words, the reaction rate decreased as the substrate concentration increased. For phenol concentrations greater than 200 g/m^3^, inhibition played an important role in the overall rate of phenol consumption.

**Table 2:** Test case conditions

$$\beta _{\rm P} = \frac{{K_{\rm P}}}{{C_{{\rm Ps}}}}\quad \beta _{{\rm PI}} = \frac{{K_{{\rm PI}}}}{{C_{{\rm Ps}}}}$$							
$$r_{{\rm PIs}} = q_{{\mathrm{max}}}\frac{{\bar X_{\rm f}}}{{Y_{\rm P}}}\left[ {\frac{{C_{{\rm Ps}}}}{{K_{\rm P} + C_{{\rm Ps}}}}} \right]\left[ {\frac{{K_{{\rm PI}}}}{{K_{{\rm PI}} + C_{{\rm Ps}}}}} \right]$$							
$$r_{{\rm PI}}^ \ast = \left( {\beta _{\rm P} + 1} \right)\left( {\beta _{{\rm PI}} + 1} \right)\frac{{C_{\rm P}^ \ast }}{{\left( {\beta _{\rm P} + C_{\rm P}^ \ast } \right)}}\frac{1}{{\left( {\beta _{{\rm PI}} + C_{\rm P}^ \ast } \right)}}$$							
$$r_{{\rm PI}}^{ \ast \prime }\left( 1 \right) = \frac{{\beta _{\rm P}}}{{\beta _{\rm P} + 1}} - \frac{1}{{\beta _{{\rm PI}} + 1}}\quad \phi ^2 = \frac{{L_{\rm f}^2}}{{\bar D_{{\rm fP}}\,C_{{\rm Ps}}}}r_{{\rm IPs}}$$							
$$I_{{\rm PI}} = \left( {\beta _{\rm P} + 1} \right)\left( {\beta _{{\rm PI}} + 1} \right){\int}_0^1 {\frac{{C_{\rm P}^ \ast }}{{\left( {\beta _{\rm P} + C_{\rm P}^ \ast } \right)}}\frac{1}{{\left[ {\beta _{{\rm PI}} + C_{\rm P}^ \ast } \right]}}} dC_{\rm P}^ \ast$$							
$$\rho _{{\rm PI}} = \left( {2\,D_{{\rm fP}}^ \ast \left( 1 \right)\,X_{\rm f}^ \ast \left( 1 \right)\,I_{{\rm PI}}} \right)^{1{\mathrm{/}}2}\quad \phi ^ \ast = \frac{\phi }{{\rho _{{\rm PI}}}}$$							
$$\sigma _{{\rm PI}} = \frac{{b\,{\mathrm{\psi }}^{\mathrm{2}}}}{{\bar X_f^2\,c\,\left( {0.2218} \right)}}\left[ {r_{{\rm PI}}^{ \ast \prime }\left( 1 \right)} \right]\,\left( F \right)\quad \sigma _{{\rm PI}}^ \ast = \sigma _{{\rm PI}}\sigma _{{\rm PI}}^2$$							
$$d_{{\rm PI}} = 1 - 2\,\sigma _{{\rm PI}}^ \ast$$							

Parameter	*C*_Ps_ (g/m^3^)						
	25	75	100	150	300	500	600
*β*_P_	0.732	0.244	0.183	0.122	0.061	0.0366	0.0305
*β*_IP_	8.58	2.86	2.145	1.43	0.715	0.429	0.3575
*r*_PI_ *’(1)	0.3183	−0.0629	−0.1633	−0.3028	−0.5256	−0.6645	−0.7071
*r*_PIs_ (g P/m^3^ h)	3133.9	3609.7	3494.12	3178.72	2381.45	1755.21	1548.8
I_PI_	0.6669	0.8446	0.9089	1.0162	1.2557	1.4867	1.5813
*ρ*_PI_	0.7799	0.8781	0.9104	0.9629	1.0701	1.1644	1.2009
*d*_PI_	0.6289	1.0930	1.2594	1.5381	2.1539	2.7271	2.9545
*ϕ* ^2^	0.2612^a^	0.1003^a^	0.0728^a^	0.0442^a^	0.0165^a^	7.31 10^-3a^	5.38 10^-3a^
	1.0446^b^	0.4011^b^	0.2912^b^	0.1766^b^	0.0662^b^	0.0293^b^	0.0215^b^
	4.1786	1.6043	1.1647	0.5755	0.2646	0.1170	0.0860
ϕ*	0.6552^a^	0.3606^a^	0.2963^a^	0.2182^a^	0.1202^a^	0.0734^a^	0.0611^a^
	1.3105^b^	0.7212^b^	0.5927^b^	0.4364^b^	0.2403^b^	0.1469^b^	0.1222^b^
	2.6210^c^	1.4424^c^	1.1854^c^	0.8729^c^	0.4807^c^	0.2938^c^	0.2443^c^
*η*	0.959^a^	1.001^a^	1.009^a^	1.012^a^	1.008^a^	1.005^a^	1.004^a^
	0.6972^b^	0.9594^b^	1.0031^b^	1.0333^b^	1.0310^b^	1.0183^b^	1.0144^b^
	0.3812^c^	0.6768^c^	0.7967^c^	0.9660^c^	1.0917^c^	1.0681^c^	1.0552^c^
*r*_ob_*(P)* (g P/m^3^ min)	50.07^a^	60.23^a^	58.73^a^	53.60^a^	40.02^a^	29.39^a^	25.91^a^
	36.42^b^	57.72^b^	58.42^b^	54.74^b^	40.92^b^	29.79^b^	26.19^b^
	19.91^c^	40.71^c^	46.40^c^	51.18^c^	43.33^c^	31.25^c^	27.24^c^
*r*_ob_*(B)* (g B/m^3^ min)	31.39^a^	37.77^a^	36.82^a^	33.61^a^	25.09^a^	18.43^a^	16.24^a^
	22.83^b^	36.19^b^	36.63^b^	34.32^b^	25.66^b^	18.68^b^	16.42^b^
	12.48^c^	25.53^c^	29.09^c^	32.09^c^	27.17^c^	19.59^c^	17.08^c^

Data corresponding to:

^a^*L*_f_ = 75 *μm*,

^b^*L*_f_ = 150 *μm*,

^c^*L*_f_ = 300 *μm*

Data without superscripts are the same for the three values of *L*_f_

*r*_IPs_ (g P/m^3^ h): rate of phenol consumption evaluated at the biofilm—fluid interface

*r*_ob_*(P)* (g P/m^3^ min): net rate of phenol consumption in the biofilm

*r*_ob_*(B)* (g B/m^3^ min): net rate of biomass production in the biofilm

The fact that the maximum growth rate was reached at lower phenol concentrations as the biofilm thickness decreased is directly related to the internal diffusion effect. This is evident from the effectiveness factor observed at different phenol concentrations and for the three biofilm thicknesses (Fig. 3b). As can be seen, *η* is almost equal to unity for a 75 μm-thick biofilm. Here the effect of internal diffusion on the net rate of phenol consumption is at a minimum. For low substrate concentrations and a thin biofilm, both effects (internal diffusion resistance and inhibition) are negligible. However, as the biofilm thickness increases, internal diffusion resistance becomes more and more important. At high substrate concentrations (greater than 200 g/m^3^), the inhibition effect becomes more important than the diffusion resistance effect, even for thick biofilms. Under these conditions, substrate diffusion resistance and inhibition negatively affect the rate of reaction and the net rate decreases with increasing *C*_Ps_.

Finally, it is interesting to note that the effectiveness factor takes on values greater than one, for the three cases where *C*_Ps_ > *200* *g/m*^*3*^. To assure that *η* will take on values greater than one (case (c)) requires:

$$\beta _{{\rm PI}} < \frac{1}{{\beta _{\rm P}}}$$

For example, when applying this condition to *C*_Ps_ = 300 g/m^3^, *β*_PI_ = 0.715 and *β*_P_ = 0.061, for any biofilm thickness one obtains:

$$0.715 < \left( {1{\mathrm{/}}0.061} \right)\,{\mathrm{or}}\,0.715 < 16.4$$

Another interesting observation regarding the behavior of *η* at different *C*_Ps_ (Fig. 3b) is the following: for each biofilm thickness, the point where *η* = 1 corresponds to a *C*_Ps_ value, where the inhibition effect is exactly compensated for by the substrate diffusion resistance.

### Effect of mass transport resistance

The substrate mass transport inside a biofilm plays an important role in the estimation of the net rate microbial growth rate. A simple analytical method to calculate this rate using a model that considers variable diffusion and activity in the biofilm is highly useful.

Recently, Connolly et al.^16^ demonstrated the influence of transport steps in biofilm growth by experimentally determining the net growth rate of *Escherichia coli* MJK2 to estimate the specific reaction rate as the net ureolysis rate per unit biofilm volume. In this case a homogeneous biofilm model was assumed and the convective external transport and diffusion rate into the biofilm were accounted for using dimensionless characteristic numbers like Damkôhler (Da) and Peclet (Pe) for external mass transport and the Thiele modulus *ϕ* (Eqs. S1–7) for diffusion into the biofilm, respectively. The reported average Da and Pe numbers of 4 and 10^4^, respectively, and the Thiele modulus of less than one suggested that urea hydrolysis was not strongly limited by either external or internal diffusion. A Michaelis–Menten rate expression was used for fitting the experimental data with the finite element method. The values of the urea kinetic expression were varied until the difference between the modeled and experimental urea fluxes was minimized. However, simple first order kinetics with respect to urea concentration was found to fit the data best. Using our semi-analytical procedure to calculate the effectiveness factor one obtains the intrinsic rate expression in a more straightforward manner. Considering Eqs. (S2–18) we calculated the effectiveness factor, taking into account the Thiele modulus values of Connolly et al.^16^ (Table 1), and the value of parameter *ρ* (Eqs. (S2–10)) for this case, which was used for normalizing the Thiele modulus. Further, the corresponding values of *η* according to our procedure for minima, maxima and average values of *ϕ* were 0.999, 0.947, and 0.998, respectively, indicating little to no internal biofilm diffusion limitation. Hence our procedure is able to estimate the effectiveness factor in experimental studies and validate conclusions on internal diffusion effects in biofilms.

### Global applicability of continuum heterogeneous biofilm model to substrate inhibition

Olivieri et al.^15^ considered a well-mixed three-phase bioreactor where the growth of a granular- *Pseudomonas* sp. OX1 biofilm was supported by the oxidation of phenol. The authors analyzed the system with either oxygen or phenol (the inhibitor) as limiting substrate. As in the present model, the empirical correlation between the substrate relative diffusivities and biomass concentration by Fan et al.^22^ was used and both the steady-state and local dynamic behavior of the system were characterized. However, as in other earlier studies a homogeneous biofilm was assumed, that is, the effective diffusivity of the substrate and the biofilm density were kept constant. The model dimensionless differential equations were solved numerically under steady-state conditions. Although the conditions in that study and the present model system were quite different, effectiveness factor and net biofilm growth rate had a similar relationship with biofilm thickness.

Similarly, *Pseudomonas stutzeri* OX1 in an airlift biofilm reactor displayed specific growth rates as a function of the initial phenol concentration that were very close to those in our test case, with a maximum growth rate at about 200 g phenol/m^3^.^14^

When modeling batch fermentation kinetics for succinic acid production by *Mannheimia succiniciproducens*, both substrate and product inhibition were considered using glucose as substrate.^23^ The growth of the microorganism was expressed by a simplified Monod model since the ratio of substrate saturation and inhibition constants was 0.0127 ≪ 1 and hence the Haldane model could be applied. The inhibition by the product was taken into account in the growth rate (1 – *C*_Pro_*/C*_ProCrit_), as suggested by Levenspiel,^24^ where *C*_Pro_ is the product concentration and *C*_ProCrit_ is the critical product concentration at which cell growth stops. The effects of initial glucose concentration on the growth rate of *M. succiniciproducens* followed a profile that would also be obtained if our procedure were applied.

In conclusion, the continuum heterogeneous biofilm model successfully predicted situations where Double Monod inhibition kinetics affects the biofilm growth rate. Analytical approximate solutions can account for limiting concentrations of substrate or inhibitor and biofilm thickness. The procedure does not require numerical simulation and calculations can be performed in a basic software package. The approach has global utility for biofilm systems of different scales ranging from microfluidic flow cells to bioreactors used in laboratories and full-scale applications where inhibition kinetics is frequently encountered. The analytical procedure is accessible to researchers from different disciplines, especially those experimenting with flow cells where substrate limitation and inhibition kinetics may exert considerable effects on biological phenomena.

## Methods

The model was designed as described in Model Development and the supplementary Information, and the experimental data used to validate the model were taken from ref. 21.

### Data availability

The authors declare that data supporting the findings of this study are available within the paper and its Supplementary Information.

### Nomenclature

$C_{\rm i}^ \ast$dimensionless substrate (*i)* concentration. Eq. (S1–4)

*C*_i_ concentration of substrate (*i*), kg/m^3^

*C*_is_concentration of substrate (*i*) at the surface of the biofilm, kg/m^3^

*d*_PI_ defined by Eq. (S2–28)

*D*_fi_ surface average effective diffusivity of substrate (*i*), m^2^/s

$D_{{\rm fi}}^ \ast$ dimensionless relative effective diffusivity of substrate (*i*), defined by Eq. (S1–4)

$\overline D _{{\rm fi}}$ average effective diffusivity of substrate (*i*) in the biofilm, m^2^/s

$\overline D _{{\rm fI}}$average effective diffusivity of inhibitor (I) in the biofilm, m^2^/s

*D*_wi_ diffusivity of substrate (*i*) in the liquid medium, m^2^/s

I_PI_ defined by Eq. (S2–17)

*K*_i_ Monod half rate constant for substrate (*i*), kg/m^3^

*K*_I_ Inhibitor concentration giving 50% inhibition rate, kg/m^3^

*L*_f_ average biofilm thickness, m

*q*_max_ maximum specific growth rate, s^−1^

*r* specific growth rate, s^−1^

*r*_b_volumetric growth rate, g biomass/(m^3^_biofilm_ ·h)

*r*_s_ reference reaction rate defined by Eq. (S1–9)

r* dimensionless rate of reaction defined by Eq. (S1–4) or (S1–14)

$r_{\rm A}^ \ast$dimensionless rate of reaction defined by Eq. (8)

$r_{\rm I}^ \ast$dimensionless rate of reaction defined by Eq. (12)

$r_{{\rm AI}}^ \ast$dimensionless rate of reaction defined by Eq. (15)

$r_{\rm A}^{ \ast \prime }$first derivative of $r_{\rm A}^ \ast$ with respect to $C_{\rm A}^ \ast$ at $C_{\rm A}^ \ast = 1$

$r_{\rm I}^{ \ast \prime }$first derivative of $r_{\rm I}^ \ast$ with respect to $C_{\rm I}^ \ast$ at $C_{\rm I}^ \ast = 1$

$r_{{\rm AI}}^{ \ast \prime }$first derivative of $r_{{\rm AI}}^ \ast$ with respect to $C_{{\rm AI}}^ \ast$ at $C_{{\rm AI}}^ \ast = 1$

*r*_iob_ average rate of substrate (*i*) consumption of the whole biofilm, kg/s m^3^

*X*_f_ biofilm density, kg/m^3^

$\overline X _f$ average biofilm density along the (*x*) direction, kg/m^3^

$X_f^ \ast$ dimensionless relative density defined by Eq. (S1–4)

*x*distance from the bottom of the biofilm, m

x*dimensionless distance defined by Eq. (S1–4)

*Y*_i_ yield coefficient for substrate (i), (kg microorganism/kg nutrient)

Greek letters

*α*_i_ effective diffusivity of substrate (i), at the bottom of the biofilm, m^2^/s

*β*_i_ dimensionless parameter for substrate (i) defined by Eq. (S1–4)

*ϕ* Thiele modulus (the ratio between a reference reaction rate in a homogeneous biofilm, which is not diffusion limited and has a density equal to the average value in the biofilm, and the diffusion rate) defined by Eq. (S1–7)

ϕ*normalized Thiele modulus, defined by Eqs. (S2–19), (S2–23) or (S2–27)

*Γ*_I_ parameter defined by Eq. (S1–8)

*Γ*_A_ parameter defined by Eq. (10)

*η* effectiveness factor (the ratio between the diffusion-limited substrate consumption rate and the substrate consumption rate that is not limited by diffusion) for a continuum heterogeneous biofilm

*η*_0_ effectiveness factor for a homogeneous biofilm

*η*_NI_ effectiveness factor for continuum heterogeneous biofilm without inhibition

*κ* parameter defined by Eq. (S1–17)

ν_i_ ratio between the substrate (*i*) yield coefficient and that of the limiting substrate

*ρ*_AI_parameter defined by Eq. (S2–16)

*σ*_A_ parameter defined by Eq. (S2–2)

*σ*_I_ parameter defined by Eq. (S2–5)

*Ψ*parameter defined by Eq. (S1–16)

Sub indexes

*i* for substrate A or inhibitory substrate I

*s*biofilm surface conditions

I for the inhibitory component

*P* for substrate phenol

## Electronic supplementary material

Supplementary information
