# Supplementary material for: Net growth rate of continuum heterogeneous biofilms with inhibition kinetics
Source: NPJ Biofilms Microbiomes. 2018 Mar 8;4:5. doi: 10.1038/s41522-017-0045-y (PMC5843665; doi:10.1038/s41522-017-0045-y)
Supplement: Supplementary file 1 — Supplementary information [file 41522_2017_45_MOESM1_ESM.doc]

**NET GROWTH RATE OF CONTINUUM HETEROGENEOUS BIOFILMS WITH INHIBITION KINETICS**

Elio Emilio Gonzo, Stefan Wuertz, and Verónica B. Rajal

**Supplementary information I**

***D_fA_(x),* *D_fI_(x),* and *X_f_(x****)* ***profiles.***

Experimental measurements of surface averaged effective diffusivity^1-4^ revealed substrate effective diffusivity to be a function of the distance (*x*) from the substratum or bottom of the biofilm that can be approximated by a linear relation. Particularly, for the limiting component *A*, the relation is:

$D_{fA}(x) = \alpha_{A} + \xi x$ (S1-1)

where *α_A_* is the effective diffusivity of substrate *A* at the bottom of the biofilm and *ξ* the effective diffusivity gradient. Both parameters depend on the substrate concentration and flow velocity at which the biofilm grew. However, there is no direct relationship between biofilm density and the coordinate (***x***) along the biofilm thickness; instead Fan et al.^5^, based on several experimental results, found the following empirical relationship between relative substrate diffusivity, *Dº_f A_*, and biofilm density:

$D_{fA}^{\mathrm{º}}(x) = 1 - \frac{0.43 X_{f}^{0.92}}{11.19 + 0.27 X_{f}^{0.99}}$ (S1-2)

The relative diffusivity of substrate *A* is the ratio between the substrate diffusivity in the biofilm and the substrate diffusivity in water:

$D_{fA}^{\mathrm{º}}(x) = \frac{D_{fA}(x)}{D_{wA}}$ (S1-3)

where *D_wA_* is the diffusivity of substrate *A* in liquid medium. It is constant and used to normalize the diffusivity of substrate A into the biofilm. *Dº_fA_(x)* is the relative diffusivity of substrate A inside the biofilm. It changes linearly along the biofilm thickness as *D_fA_(x)*, as demonstrated elsewhere^1-4^. The higher the density of the biofilm, the less open volume is available to the substrate to diffuse through the biofilm. Note that Equation (S1-2) has been validated for a limited range of biofilm reactor systems under specific operating conditions. It should not be interpreted as a basic law applicable to any biofilm or reactor condition per se and may be replaced with a more general equation in future work. To generalize the treatment of the problem we used dimensionless variables as shown in Eq. (S1-4) below.

***Dimensionless equations for the system***

The dimensionless distance (*x^*^*), concentrations ($C_{A}^{*}$and$C_{I}^{*}$), reaction rate (*r^*^*), relative effective diffusivities (*D^*^_fA_* and*D^*^_fI_*), relative density (*X^*^_f_*), and the Double-Monod half rate constants *β_A_* and *β_I_*, dimensionless parameters for substrate *A* and inhibitor *I*, can be defined as:

$$x* = \frac{x}{L_{f}}$$

$$C_{A}^{*} = \frac{C_{A}}{C_{As}}$$

$$C_{I}^{*} = \frac{C_{I}}{C_{Is}}$$

$$\beta_{A} = \frac{K_{A}}{C_{As}}$$

$$\beta_{I} = \frac{K_{I}}{C_{Is}}$$

(S1-4)

$$r^{*} =\frac{r}{r_{s}}$$

$$D_{fA}^{*} = \frac{D_{fA}}{\overline{D}_{fA}}$$

$$D_{fI}^{*} = \frac{D_{fI}}{\overline{D}_{fI}}$$

$$X_{f}^{*} = \frac{X_{f}}{\overline{X}_{f}}$$

where parameters $\overline{D}_{fA}$*,*$\overline{D}_{fI}$ are the average effective diffusivities of substrate *A* and inhibitor *I*, respectively, and $\overline{X}_{f}$is the average biofilm density along the biofilm thickness.

Assuming species *A* as the limiting substrate and introducing the dimensionless parameters in Equations (2) and (3), the following differential equations are found:

$\frac{d}{dx*} D_{fA}^{*} \frac{dC_{A}^{*}}{dx*} = \varphi^{2} X_{f}^{*} r^{*}(C_{A}^{*},C_{I}^{*})$ (S1-5)

$\frac{d}{dx*} D_{fI}^{*} \frac{dC_{I}^{*}}{dx*} = \varphi^{2} \Gamma_{I} X_{f}^{*} r^{*}(C_{A}^{*},C_{I}^{*})$ (S1-6)

Where the Thiele modulus *φ* is:

$\varphi^{2} = \frac{L_{f}^{2}}{\overline{D}_{fA} C_{As}} \frac{q_{\max}}{Y_{A}} \overline{X}_{f} \left(\frac{C_{As}}{K_{A}+ C_{As}}\right)\left(\frac{K_{I}}{K_{I}+ C_{Is}}\right) =\frac{L_{f}^{2} \overline{X}_{f}}{\overline{D}_{fA} C_{As} Y_{A}} r_{s}$ (S1-7)

and $\Gamma_{I} = \nu_{I} \frac{C_{As} \overline{D}_{fA}}{C_{Is} \overline{D}_{fI}}$ with $\nu_{I} = \frac{\gamma_{A}}{\gamma_{I}}\frac{Y_{A}}{Y_{I}}$ (S1-8)

$r_{s} = q_{\max} \left(\frac{C_{As}}{K_{A}+ C_{As}}\right)\left(\frac{K_{I}}{K_{I}+ C_{Is}}\right)$ (S1-9)

The relation between the stoichiometric and yield coefficients of species *A* and *I* is *ν_I_.* Therefore, *ν_I_* will be positive if *I* is a reactant and negative if it is a reaction product. The dimensionless boundary conditions, for the differential equations (S1-5) and (S1-6), are:

At $x^{*} = 1$ $C_{A}^{*} = 1$ $C_{I}^{*} = 1$ (S1-10)

At $x^{*} = 0$ $\frac{dC_{A}^{*}}{dx^{*}} = 0$ $\frac{dC_{I}^{*}}{dx} = 0$ (S1-11)

Beyenal and Lewandowski^2^ have found that the variation in effective diffusivity across the biofilm is constant. We assume that this diffusivity gradient *ξ* is constant for any of the substrates since it is related to the biofilm heterogeneity^6^. Therefore, the ratios between $\overline{D}_{fA}$and $\overline{D}_{fI}$, and between $D_{fA}$ and $D_{fI}$, remain almost constant. Consequently:

$D_{fA}^{*}(x^{*}) \cong D_{fI}^{*}(x^{*})$ (S1-12)

By taking into account equation (S1-12) and dimensionless boundary conditions (S1-10) and (S1-11), the relationship between *C_A_^*^* and *C_I_^*^* is found by solving equations (S1-5) and (S1-6):

$C_{I}^{*} = \Gamma_{I} (C_{A}^{*}-1)+ 1$ (S1-13)

For substrate *A* to be the limiting component, *Γ_I_* should be lower than one, if *I* is a reactant. In this case, the concentration profiles in the biofilm will be those shown in Figure 1.

According to the definition of dimensionless parameters (S1-4) and the specific growth rate (1), the dimensionless kinetic expression *r^*^*, is:

$r^{*} = (\beta_{A}+ 1)(\beta_{I}+ 1)\frac{C_{A}^{*}}{(\beta_{A}+ C_{A}^{*})} \frac{1}{(\beta_{I}+C_{I}^{*})}$ (S1-14)

Therefore, the dimensionless differential equation (S1-5), considering the relation (S1-13), will be:

$\frac{d}{dx*} D_{fA}^{*} \frac{dC_{A}^{*}}{dx*} = \varphi^{2} X_{f}^{*} r^{*}(C_{A}^{*})$ (S1-15)

To solve differential equation (S1-15) it is necessary to know the relationship between$D_{fA}^{*}$ and$X_{f}^{*}$ with the dimensionless coordinate$x^{*}$.

Defining the dimensionless parameters:

$\Psi= \frac{\alpha_{A}}{L_{f} \xi}$ (S1-16) and $\kappa= \frac{D_{w}}{\alpha_{A}}$ (S1-17)

Following the procedure presented by Beyenal and Lewandowski^4^, and considering that the average effective diffusivity in the biofilm, $\overline{D}_{fA}$, is equal to

$\overline{D}_{fA} = \alpha_{A} + \frac{\xi L_{f}}{2}$, (S1-18)

Beyenal and Lewandowski^4^ found the needed relation between the dimensionless effective diffusivity and biofilm density as a function of *x**:

$D_{fA}^{*}(x^{*}) = \frac{2 (\Psi+x^{*})}{2 \Psi+1} = c \left(1+\frac{x^{*}}{\Psi}\right)$ (S1-19)

and

$X_{f}^{*}(x^{*}) =\frac{-38.856+38.976 \kappa^{0.7782} \left(1+\frac{x^{*}}{\Psi}\right)^{- 0.7782}}{\overline{X}_{f}} =\frac{a+b \left(1+\frac{x^{*}}{\Psi}\right)^{- 0.7782}}{\overline{X}_{f}}$ (S1-20)

To summarize, Beyenal and Lewandowski^2^ gave the values of the constants in Eq. (S1-1) for a specific case that we assumed and applied to solve the set of dimensionless differential equations (S1-5) and (S1-6). Equation (S1-2) is the general relation between diffusion coefficients and biofilm density proposed by Fan et al.^5^. Since it is necessary to know the relationship between *D^*^_fA_* and *X^*^_f_* with the dimensionless coordinate *x^*^*, Eq. (S1-19) and (S1-20) were found from the earlier ones. For the specific test case study presented here, Eq. (S1-19) and (S1-20) are Eq. (26) and (27) in the accompanying manuscript.

**Effectiveness factor estimation**

The effectiveness factor *η*, is the ratio between the observed limiting substrate consumption rate, *r_Aob_* (rate of reaction with diffusion resistance), and the substrate consumption rate without diffusion limitation^7^, as given by Eq. (S1-21):

$\eta= \frac{r_{Aob}}{r_{As}} = \int_{0}^{1} X_{f}^{*} r^{*}(C_{A}^{*}) dx^{*}$ (S1-21)

Thus, the actual observed reaction rate (net rate of substrate consumption) could be directly obtained from Eq. (S1-21).

In addition, taking into account the definition of *η*, the limiting substrate steady state mass balance at the biofilm-fluid interphase, gives:

$D_{fA}(x=L_{f}) \frac{dC_{A}}{dx}\left|_{x=L_{f}}\right. S_{x} = \eta\frac{q_{\max}}{Y_{A} } \frac{C_{As}}{(K_{A}+ C_{As})} \frac{K_{I}}{(K_{I}+ C_{Is})} S_{x} L_{f}$ (S1-22)

where *S_x_* is the biofilm-fluid interphase area.

Considering the dimensionless variables and parameters previously defined, Eq. (S1-22) yields:

$\frac{D_{fA}^{*}(1)}{\varphi^{2}} \left(\frac{dC_{A}^{*}}{dx^{*}}\right)_{x^{*}=1} = \eta$ (S1-23)

Equations (S1-21) and (S1-23) can be used to obtain an expression for *η*. However, since *C^*^_A_*(*x^*^*) is not known, *η* cannot be estimated. Nevertheless, Eq. (S1-15) can be solved approximately by a perturbation procedure^8^, when $\varphi<< 1$ or when $\varphi>> 1$, and using a matching expression to find the analytical equation for *η*, valid for the entire range of $\varphi$values. This procedure was used successfully in estimating the effectiveness factor for a continuum heterogeneous biofilm with both single- and multiple-substrate Monod kinetics^9,6^.

**Supplementary information II**

**Perturbation solutions to case study**

The application of the perturbation and matching technique to obtain the general solution for *η* versus *φ* for the system studied here is given following the procedure previously presented^9^.

**When *φ ^2^ <<* 1**, the perturbation solution for *η* is a series with terms up to the order of *φ^2^*, given by:

**Case (a):**

$\eta= 1 - \sigma_{A} \varphi^{2}$ (S2-1)

With

$\sigma_{A} = \frac{ b \Psi^{2}}{\overline{X}_{f}^{2} c (0.2218)} [r_{A}^{* '}(1)] \left(F\right)$ (S2-2)

Where

$$F= - \frac{ a \Psi}{(0.2218)(1.2218)}\left[\left(1+\frac{1}{\Psi}\right)^{1.2218}- 1\right] + \frac{ a \left(1+\frac{1}{\Psi}\right)^{0.2218}}{ (0.2218)} -$$

$$- a \int_{0}^{1} \ln\left[\frac{1+\frac{1}{\Psi}}{1+\frac{x^{*}}{\Psi}}\right] dx^{*} - \frac{ b \Psi\left[\left(1+\frac{1}{\Psi}\right)^{0.4436} - 1\right]}{ (0.2218) (0.4436)} +$$

$$+ \frac{b \Psi\left(1+\frac{1}{\Psi}\right)^{0.2218}}{ (0.2218)^{2}}\left[\left(1+\frac{1}{\Psi}\right)^{0.2218}- 1\right] - b \int_{0}^{1} \left(1+\frac{x^{*}}{\Psi}\right)^{- 0.7782} \ln\left[\frac{1+\frac{1}{\Psi}}{1+\frac{x^{*}}{\Psi}}\right] dx^{*}$$

(S2-3)

**Case (b):**

$\eta= 1 - \sigma_{I} \varphi^{2}$ (S2-4)

Where $\sigma_{I} = \frac{ b \Psi^{2}}{\overline{X}_{f}^{2} c (0.2218)} [r_{I}^{* '}(1)] \left(F\right)$ (S2-5)

**Case (c):**

$\eta= 1 - \sigma_{AI} \varphi^{2}$ (S2-6)

With $\sigma_{AI} = \frac{ b \Psi^{2}}{\overline{X}_{f}^{2} c (0.2218)} [r_{AI}^{* '}(1)] \left(F\right)$ (S2-7)

With *F* in equations (S2-2), (S2-5) and (S2-7) given by equation (S2-3).

*F* is always the same function for any case, since it depends only on the degree of biofilm heterogeneity, *ξ*.

**When *φ ^2^ → ∞***, the reaction rate is very fast and diffusion is low; therefore, the limiting substrate is completely consumed at the biofilm-fluid interphase. The dimensionless biofilm density and substrate effective diffusivity take on a value corresponding to position *x* =* 1:

$X_{f}^{*}(x*) = X_{f}^{*}(1)$ ; $D_{fA}^{*}(x*) = D_{fA}^{*}(1)$ and $D_{fI}^{*}(x*) = D_{fI}^{*}(1)$ (S2-8)

Consequently, depending on which is the limiting component, Eqs. (18) to (20) yield:

$$D_{fA}^{*}(1) \frac{d^{2}C_{A}^{*}}{dx*^{2}} = \varphi^{2} X_{f}^{*}(1) r_{A}^{*}$$

$D_{fI}^{*}(1) \frac{d^{2}C_{I}^{*}}{dx*^{2}} = \varphi^{2} X_{f}^{*}(1) r_{I}^{*}$ (S2-9)

$$D_{fA}^{*}(1) \frac{d^{2}C_{A}^{*}}{dx*^{2}} = \varphi^{2} X_{f}^{*}(1) r_{AI}^{*}$$

Following the procedure presented in Gonzo et al.^9^, the solution for each case is found.

**Case (a):**

$\eta= \frac{\left(2 D_{fA}^{*}(1) X_{f}^{*}(1) I_{A}\right)^{ 1/2}}{\varphi} = \frac{\rho_{A}}{\varphi}$ (S2-10)

Where,

$\rho_{A} = \left(2 D_{fA}^{*}(1) X_{f}^{*}(1) I_{A}\right)^{1 / 2}$ (S2-11)

And

$I_{A} = (\beta_{A}+1) (\beta_{I}+1) \int_{0}^{1} \frac{C_{A}^{*}}{(\beta_{A}+C_{A}^{*})} \frac{1}{[\beta_{I}+\Gamma_{I}(C_{A}^{*}-1)+1]} dC_{A}^{*}$ (S2-12)

It must be taken into account that when$C_{A}^{*}=0$, the reaction rate goes to zero.

**Case (b):**

If the inhibitor is the limiting component, when$C_{I}^{*}=0$, there is no more inhibition effect on the reaction rate. According to Eq. (12), at this point $C_{A}^{*}= 1-\Gamma_{A}$ and the reaction continues. Therefore, in the calculation of parameter *ρ_I_*, the kinetics of the reaction rate is equal to that given by Eq. (12) between $C_{I}^{*}=1$ and $C_{I}^{*}=0$, and equal to a Monod kinetic expression for a single substrate (depending only on the concentration of substrate *A*), between $C_{A}^{*}= 1-\Gamma_{A}$ and $C_{A}^{*}=0$. It follows that

$\eta= \frac{\left(2 D_{fI}^{*}(1) X_{f}^{*}(1) I_{I}\right)^{ 1/2}}{\varphi} = \frac{\rho_{I}}{\varphi}$ (S2-13)

with

$$I_{I} = (\beta_{A}+1) (\beta_{I}+1) \int_{0}^{1} \frac{[\Gamma_{A}(C_{I}^{*}-1)+1]}{[\beta_{A}+\Gamma_{A}(C_{I}^{*}-1)+1]}\frac{1}{(\beta_{B}+C_{I}^{*})} dC_{I}^{*} +$$

$+ (\beta_{A}+1) \int_{0}^{1-\Gamma_{A}} \frac{C_{A}^{*}}{(\beta_{A}+C_{A}^{*})} dC_{A}^{*}$ (S2-14)

**Case (c):**

$\eta= \frac{\left(2 D_{fA}^{*}(1) X_{f}^{*}(1) I_{AI}\right)^{ 1/2}}{\varphi} = \frac{\rho_{AI}}{\varphi}$ (S2-15)

$\rho_{AI} = \left(2 D_{fA}^{*}(1) X_{f}^{*}(1) I_{AI}\right) ^{1/2}$ (S2-16)

$I_{AI} = (\beta_{A}+1) (\beta_{AI}+1) \int_{0}^{1} \frac{C_{A}^{*}}{(\beta_{A}+C_{A}^{*})} \frac{1}{(\beta_{AI}+C_{A}^{*})} dC_{A}^{*}$ (S2-17)

***The matching equation***

The matching equation proposed by Gonzo et al.^9^ is also effective in matching asymptotic expressions (S2-1) and (S2-10) for case (a); Eq. (S2-4) and (S2-13) for case (b), and Eq. (S2-6) and (S2-15) for case (c).

**Case (a):**

$\eta= \left[\varphi^{*2} + \exp(- d_{A} \varphi^{*2})\right]^{ - (1/2)}$ (S2-18)

With

$\varphi^{*} = \frac{\varphi}{\rho_{A}}$ (S2-19)

$d_{A} = 1 - 2 \sigma_{A}^{*}$ (S2-20)

and

$\sigma_{A}^{*} = \sigma_{A} \rho_{A}^{2}$ (S2-21)

For the scenario where the parameter *d_A_* is less than zero, it is set as *d_A_ =* 0.

**Case (b):**

$\eta= \left[\varphi^{*2} + \exp(- d_{I} \varphi^{*2})\right]^{ - (1/2)}$ (S2-22)

With

$\varphi^{*} = \frac{\varphi}{\rho_{I}}$ (S2-23)

$d_{I} = 1 - 2 \sigma_{I}^{*}$ (S2-24)

and

$\sigma_{I}^{*} = \sigma_{I} \rho_{I}^{2}$ (S2-25)

For the scenario where the parameter *d_I_* is less than zero, it is set as *d_I_ =* 0.

**Case (c):**

The general solution for the effectiveness factor will be given by:

$\eta= \left[\varphi^{*2} + \exp(- d_{I} \varphi^{*2})\right]^{ - (1/2)}$ (S2-26)

with

$\varphi^{*} = \frac{\varphi}{\rho_{AI}}$ (S2-27) $d_{AI} = 1 - 2 \sigma_{AI}^{*}$ (S2-28)

and

$\sigma_{AI}^{*} = \sigma_{AI} \rho_{AI}^{2}$ (S2-29)

For the scenario where the parameter *d_AI_* is less than zero, it is set as *d_AI_ =* 0.

**NOMENCLATURE**

*a* parameter defined by Eq. (S1-20), kg/m^3^

*b* parameter defined by Eq. (S1-20), kg/m^3^

*C_i_** dimensionless substrate (*i)* concentration. Eq. (S1-4)

*C_i_* concentration of substrate (*i*), kg/m^3^

*C_is_* concentration of substrate (*i*) at the surface of the biofilm, kg/m^3^

*c* dimensionless parameter defined by Eq. (S1-19)

*D_f i_* surface average effective diffusivity of substrate (*i*), m^2^/s

*D*_f i_* dimensionless relative effective diffusivity of substrate (*i*), defined by Eq. (S1-4)

average effective diffusivity of substrate (*i*) in the biofilm, m^2^/s

average effective diffusivity of inhibitor (*I*) in the biofilm, m^2^/s

*Dº_f i_* relative effective diffusivity of substrate (*i*), defined by Eq. (S1-3)

*D_wi_* diffusivity of substrate (*i*) in the liquid medium, m^2^/s

*d_A_* dimensionless parameter defined by Eq. (S2-20)

*d_I_* dimensionless parameter defined by Eq. (S2-24)

*d_AI_* dimensionless parameter defined by Eq. (S2-28)

*F* function defined by Eq. (S2-3)

*I_A_* parameter defined by Eq. (S2-12)

*I_I_* parameter defined by Eq. (S2-14)

*I_AI_* parameter defined by Eq. (S2-17)

*K_i_* Monod half rate constant for substrate (*i*), kg/m^3^

*K_I_* Inhibitor concentration giving 50% inhibition rate, kg/m^3^

*L_f_* average biofilm thickness, m

*q_max_* maximum specific growth rate, s^-1^

*r* specific growth rate, s^-1^

*r_s_* reference reaction rate defined by Eq. (S1-9)

dimensionless rate of reaction defined by Eq. (S1-4) or (S1-14)

dimensionless rate of reaction defined by Eq. (8)

dimensionless rate of reaction defined by Eq. (12)

dimensionless rate of reaction defined by Eq. (15)

first derivative of respect to at

first derivative of respect to at

first derivative of respect to at

*r_iob_* average rate of substrate (*i*) consumption of the whole biofilm, kg/s m^3^

*S_x_* biofilm-fluid interphase surface area, m^2^

*X_f_* biofilm density, kg/m^3^

average biofilm density along the (*x*) direction, kg/m^3^

*X*_f_* dimensionless relative density defined by Eq. (S1-4)

*x* distance from the bottom of the biofilm, m

*x** dimensionless distance defined by Eq. (S1-4)

*Y_i_* yield coefficient for substrate (*i*), (kg microorganism/kg nutrient)

*Greek letters*

*α_i_* effective diffusivity of substrate (*i*), at the bottom of the biofilm, m^2^/s

*β_i_* dimensionless parameter for substrate (*i*) defined by Eq. (S1-4)

*φ* Thiele modulus, Eq. (S1-7)

*φ** normalized Thiele modulus, defined by Eq. (S2-19), (S2-23) or (S2-27)

*γ_i_* stoichiometric coefficient of species *i*

*Γ_A_* parameter defined by Eq. (10)

*Γ_I_* parameter defined by Eq. (S1-8)

*η* effectiveness factor for a continuum heterogeneous biofilm

*κ* parameter defined by Eq. (S1-17)

*ν_i_* ratio between the substrate (*i*) yield coefficient and that of the limiting substrate

*ρ_A_* parameter defined by Eq. (S2-11)

*ρ_I_* parameter defined by Eq. (S2-13)

*ρ_AI_* parameter defined by Eq. (S2-16)

*σ_A_* parameter defined by Eq. (S2-2)

*σ_I_* parameter defined by Eq. (S2-5)

*σ_AI_* parameter defined by Eq. (S2-7)

*σ_A_^*^* parameter defined by Eq. (S2-21)

*σ^*^_AI_* parameter defined by Eq. (S2-29)

parameter defined by Eq. (S2-25)

- effective diffusivity gradient, m/s

*Ψ* parameter defined by Eq. (S1-16)

*Sub indexes*

*i* for substrate *A* or inhibitory substrate *I*

*s* biofilm surface conditions

*I* for the inhibitory component

*P* for substrate phenol

**REFERENCES**

1. Beyenal H, Tanyolac A, Lewandowski Z. Measurement of local effective diffusivity in heterogeneous biofilms. *Water Sci Technol* **38:** 171-178 (1998).
2. Beyenal H, Lewandowski Z. Combined effect of substrate concentration and flow velocity on effective diffusivity in biofilms. *Water Res* **34:** 528-538 (2000)
3. Beyenal H, Lewandowski Z. Internal and external mass transfer in biofilms grown at various flow velocities. *Biotechnol Prog* **18:** 55-61 (2002).
4. Beyenal H, Lewandowski Z. Modeling mass transport and microbial activity in stratified biofilm. *Chem Eng Sci* **60:** 4337-4348 (2005).
5. Fan LS, Leyva-Ramos R, Wisecarver KD, Zehner BJ. Diffusion of phenol through a biofilm grown on activated carbon particles in a draft-tube 3-phase fluidized-bed bioreactor. *Biotechnol Bioeng* **35:** 279-286 (1990).
6. Gonzo EE, Wuertz S, Rajal VB. The continuum heterogeneous biofilm model with multiple limiting substrate Monod kinetics. *Biotechnol Bioeng* **111:** 2252-2264 (2014).
7. Scott Fogler H. Elements of Chemical Reaction Engineering. Prentice-Hall: New Jersey, US, 1999.
8. Gonzo EE, Gottifredi JC. A simple and accurate method for simulation hollow fiber biocatalyst membrane reactors. *Biochem Eng J* **37**: 80-85 (2007).
9. Gonzo EE, Wuertz S, Rajal VB. Continuum heterogeneous biofilm model – A simple and accurate method for effectiveness factor determination. *Biotechnol Bioeng* **109**(7): 1779-1790 (2012).
